# Detection of PPB-Level H_2_S Concentrations in Exhaled Breath Using Au Nanosheet Sensors with Small Variability, High Selectivity, and Long-Term Stability

**DOI:** 10.1021/acssensors.3c01944

**Published:** 2024-02-09

**Authors:** Taro Kato, Takahisa Tanaka, Ken Uchida

**Affiliations:** Department of Materials Engineering, The University of Tokyo, Tokyo 113-8656, Japan

**Keywords:** gas sensor, hydrogen sulfide, gold nanosheet, breath analysis, ppb-level detection

## Abstract

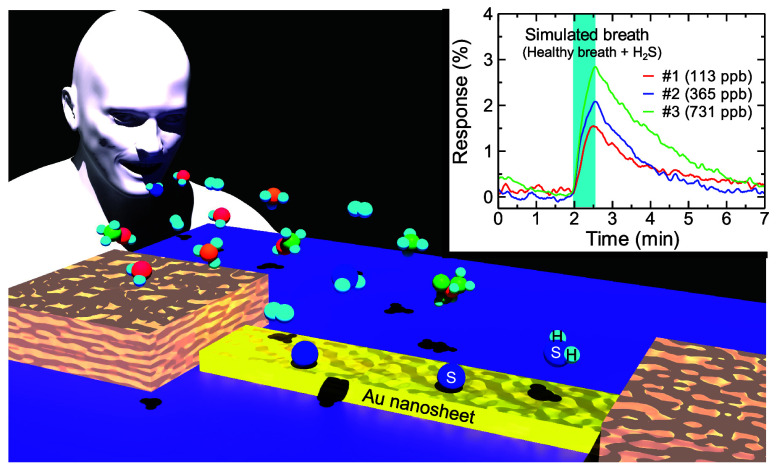

The continuous monitoring of hydrogen sulfide (H_2_S) in exhaled breath enables the detection of health issues such as halitosis and gastrointestinal problems. However, H_2_S sensors with high selectivity and parts per billion-level detection capability, which are essential for breath analysis, and facile fabrication processes for their integration with other devices are lacking. In this study, we demonstrated Au nanosheet H_2_S sensors with high selectivity, ppb-level detection capability, and high uniformity by optimizing their fabrication processes: (1) insertion of titanium nitride (TiN) as an adhesion layer to prevent Au agglomeration on the oxide substrate and (2) N_2_ annealing to improve nanosheet crystallinity. The fabricated Au nanosheets successfully detected H_2_S at concentrations as low as 5.6 ppb, and the estimated limit of detection was 0.5 ppb, which is superior to that of the human nose (8–13 ppb). In addition, the sensors detected H_2_S in the exhaled breath of simulated patients at concentrations as low as 175 ppb while showing high selectivity against interfering molecules, such as H_2_, alcohols, and humidity. Since Au nanosheets with uniform sensor characteristics enable easy device integration, the proposed sensor will be useful for facile health checkups based on breath analysis upon its integration into mobile devices.

Health checkups based on breath testing are attracting attention.^1,2^ Exhaled breath contains more than 200 types of chemical species, and the kinds and amounts of these species give information about various diseases.^3−5^ Hence, the continuous evaluation of the gas components of exhaled breath enables the detection of diseases, even in presymptomatic states.^6^ Exhaled breath contains hydrogen sulfide (H_2_S) molecules produced by sulfate-reducing bacteria at concentrations of less than 1000 parts per billion (ppb).^7,8^ H_2_S is closely related to various health issues, such as halitosis, gum disease, and gastrointestinal problems.^9,10^ Exhaled breath contains abundant reducing gases other than H_2_S, with the concentration of H_2_S being much lower than those of these other reducing gases (e.g., 1–100 ppm for hydrogen (H_2_)).^11,12^ Thus, breath monitoring requires the development of H_2_S sensors with fast responses, parts per billion-level limits of detection (LODs), and high selectivity against interfering gases in exhaled breath.

Various types of H_2_S sensors have been proposed and used to address these requirements.^13,14^ The most common H_2_S sensors are based on semiconducting metal oxides; they have low costs and are operated rapidly and easily.^15−17^ However, metal oxide semiconductor (MOS) gas sensors typically have poor selectivity because they react to all reducing or oxidizing gases.^18,19^ Electrochemical sensors are also commercialized, but they are less sensitive than MOS gas sensors and susceptible to temperature and humidity changes.^20,21^ Other types of H_2_S sensors have been proposed, such as colorimetric sensors,^22^ surface acoustic wave gas sensors,^23^ and sensors made using conductive materials (e.g., graphenes and polymers).^24,25^ However, these sensors are hindered by challenges related to selectivity or ppb-level detection. Decorating conducting materials, such as MOS,^16,26^ graphenes,^24^ and carbon nanotubes (CNTs),^27^ with metal and MOS nanoparticles (NPs) has recently been used to achieve sensor selectivity and ppb-level detection. However, the complicated nature of these decoration processes hinders the fabrication of H_2_S sensors with consistent characteristics and the integration of these sensors with other chemical sensors. In summary, to the best of our knowledge, a H_2_S sensor with high selectivity, parts per billion-level detection capability, and a simple fabrication process that yields uniform sensor characteristics has not been developed.

In this study, we utilized metal nanosheets as gas sensors.^28,29^ The sensing mechanism of the metal nanosheet gas sensor is that the chemisorption of gas molecules on the metal surface changes the nanosheet resistance, while the physisorption hardly affects its resistance.^30−32^ Since the chemisorption is based on the catalytic reaction, it can be highly selective to the specific gas molecules by properly selecting the metals.^28^ Furthermore, in a metal nanosheet gas sensor, the metal itself works not only as a receptor of the target gas but also as a transducer of chemisorption phenomena into electrical signals. These sensors differ from NP-functionalized sensors, which use additional transducers such as oxide nanowires and graphenes. Since metal nanosheet gas sensors are fabricated using simple lithographic processes, their sizes and shapes can be made uniform, which is advantageous for the integration of sensors.^28^

We chose gold (Au) as the sensing material because it is the noblest of all the metals,^33^ which is preferred for the selectivity but has a strong affinity for sulfur-containing compounds.^34^ Although H_2_S has been detected using Au films by several researchers,^35−39^ the selectivity, ppb-level detection capability, fast response–recovery characteristics, and breath measurement performance of their sensors were not demonstrated.

Here, we created Au nanosheet H_2_S sensors with high uniformity, ppb-level detection capability, and fast response–recovery characteristics by optimizing their fabrication process. We adopted titanium nitride (TiN) as an adhesion layer^40^ to prevent Au’s agglomeration and interdiffusion with the other elements. The Au nanosheets are annealed to improve their crystallinity characteristics. The fabricated Au nanosheet sensors showed good selectivity, ppb-level detection capability, and response–recovery characteristics and successfully estimated H_2_S concentrations in breath samples from simulated patients (simulated breaths); these simulated breaths were generated by injecting small amounts of H_2_S into healthy breaths. We also performed surface analysis and first-principles density functional theory (DFT) calculations to explain the sensing mechanism of the sensors. Findings suggested that the atomically adsorbed sulfur forms projected densities of states (PDOSs) near the Fermi level of Au, and these states induce changes in the resistance of the Au nanosheet. The developed H_2_S sensors can be easily implemented in mobile devices, thus helping realize daily health checkups.

## Experimental Section

### Fabrication Strategy of Au Nanosheet

**Figure 1:**
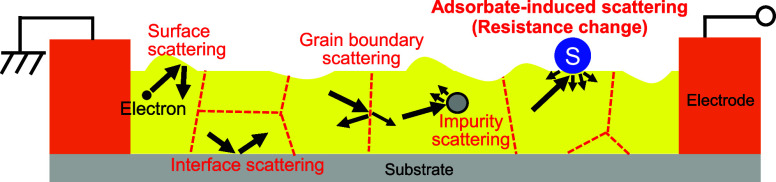
Schematic of scattering factors in a metal nanosheet. The additional molecules adsorbed on the nanosheet change its resistance.

The following electron scattering factors determine the resistance of a metal nanosheet: electron–electron scattering,^41^ electron–phonon scattering,^42^ surface (interface) scattering,^42^ grain boundary scattering,^42^ impurity scattering,^42^ and adsorbate-induced scattering^30^ (Figure 1). Since the electron–electron scattering and the electron–phonon scattering are intrinsic to the metal material and its thickness,^41,43^ it is impossible to suppress these effects. The other scattering factors depend on the morphology of the nanosheet,^42,44,45^ and these factors are controllable. Because the amount of adsorbate-induced scattering determines sensor response,^30^ this scattering factor should be maximized while suppressing the other scattering factors (surface scattering, grain boundary scattering, and impurity scattering). Thus, a high-crystallinity nanosheet is necessary.

Nanosheet resistance changes caused by molecular adsorption on a metal nanosheet increase as the nanosheet becomes thinner.^46,47^ Thus, thinner nanosheets are important for an improved sensor response. However, the structural stability of thin-film metals typically decreases with their thickness, and agglomeration occurs at low temperatures.^48^ Agglomeration must be suppressed to prevent structural variabilities and resistivity increases, which compromise the sensor response.

**Figure 2:**
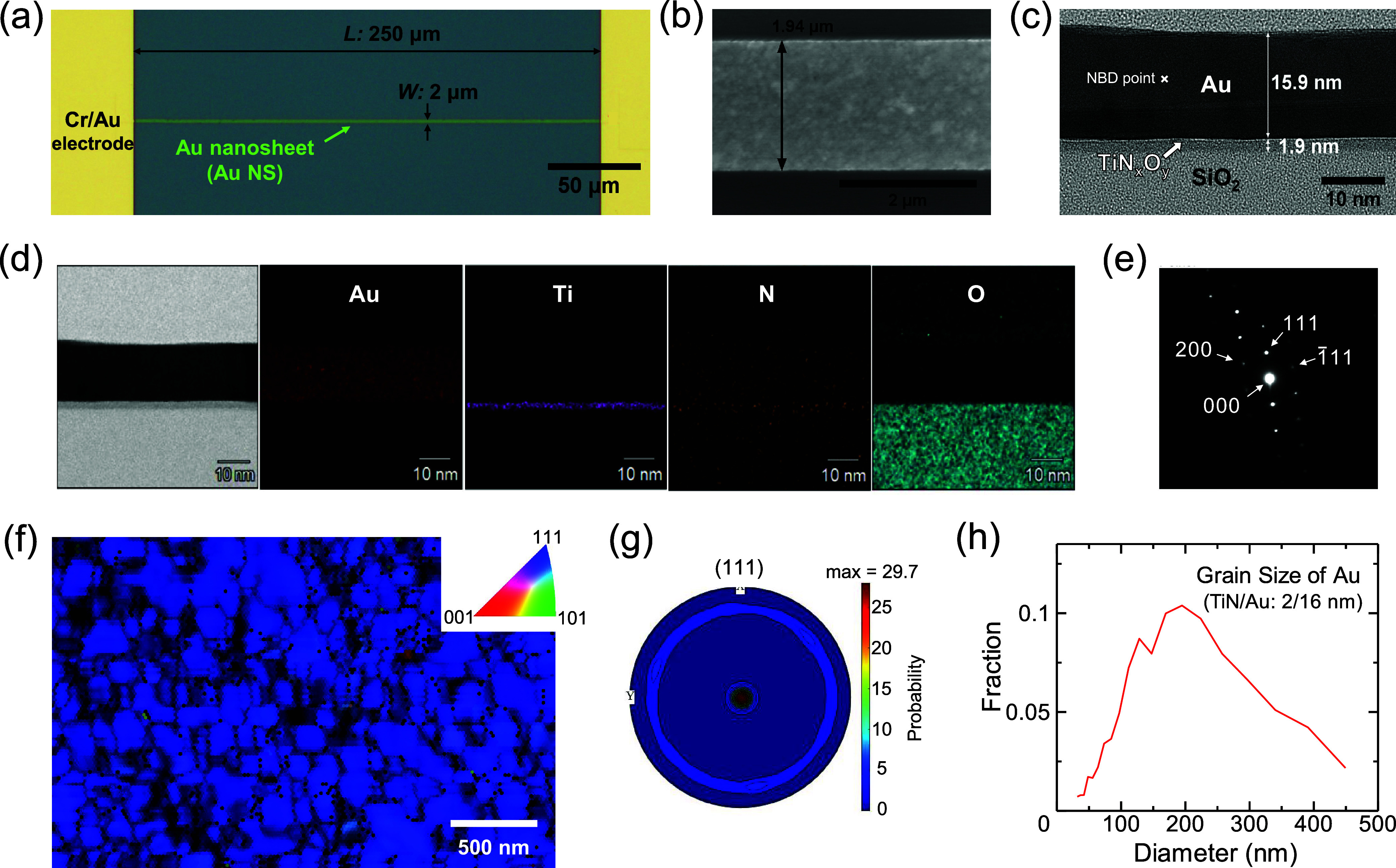
(a) Optical image of the Au nanosheet with Cr/Au electrodes. (b) SEM image of the Au nanosheet. (c) Cross-sectional TEM image of the Au nanosheet. The location of the NBD measurement in (e) is also shown. (d) Elemental distributions of Au, Ti, N, and O (from EDX). (e) Diffraction pattern obtained from NBD with indications of surface orientations. (f) EBSD IPF map. (g) EBSD PF of the Au nanosheet showing crystallographic orientations of grains. (h) Grain size distribution of the Au nanosheet (from the EBSD map).

Au possesses weak adhesion characteristics on the oxide substrates. Some voids start to form in Au films with thicknesses of 20 nm on silicon dioxide (SiO_2_) at 200 °C.^49^ Insertion of an adhesion layer can suppress agglomeration. Chromium (Cr) and titanium (Ti) are usually utilized as adhesion layer elements between Au and SiO_2_.^40^ However, these materials diffuse into Au up to the film surface at temperatures above 150 °C and form oxide layers.^50^ These impurities and oxide layers increase the resistance of a Au nanosheet and considerably degrade the sensor response. Thus, a material that achieves good adhesion and does not diffuse into Au is needed as an adhesion layer. We chose TiN as the adhesion layer in this study. TiN has good adhesion characteristics with Au,^40^ and interdiffusion is prevented by the strong bonding energy between Ti and nitrogen (N). Figure S1a in the Supporting Information shows the temperature dependence of the resistivity of 12 nm-thick Au nanosheets with different 2 nm-thick adhesion layers: Cr, Ti, and TiN. The resistivity of the Au nanosheet with the Cr adhesion layer significantly increased compared to that of the as-deposited Au nanosheet. This indicated that Cr diffused into the Au nanosheet and increased the impurity scatterings. The resistivity of the Au nanosheet with the Ti adhesion layer slightly decreased after annealing. This implied that the annealing slightly improved its crystallinity, or the effect of decreasing the grain boundary scatterings and the effect of increasing the impurity scatterings were competing. On the other hand, the resistivity of the Au nanosheet with the TiN adhesion layer remarkably decreased by annealing (Figure S1b), indicating that the grain boundary scatterings remarkably decreased. Furthermore, the elemental distribution in Figure 2d clarified that TiN did not diffuse into Au. Therefore, we determined TiN to be the best adhesion layer of the Au nanosheets.

In addition to the adhesion layer, the annealing condition was optimized. Because Au nanosheet devices need to be operated at temperatures above 200 °C for rapid recovery, as described in the Gas-Sensing Characteristics section, they should be preannealed at temperatures higher than 200 °C to improve their crystallinity characteristics (decrease their grain boundaries) and prevent atomic diffusion during operation. Figure S2 shows the optical microscopic images of the surfaces of the 16 nm-thick Au nanosheets with the 2 nm-thick TiN adhesion layers annealed at different temperatures. Numerous voids started to form above 400 °C, while no voids appeared in the Au nanosheet annealed at 300 °C. Therefore, we determined the optimal annealing temperature to be 300 °C.

### Fabrication of Au Nanosheet

A schematic diagram of the Au nanosheet fabrication process is shown in Figure S3. We deposited a Au nanosheet (16 nm thickness) with a 2 nm TiN adhesion layer on a silicon (Si) substrate with a thermally grown SiO_2_ layer (1 μm thickness). The TiN and Au layers were formed via sputtering (CFS-4ES, Shibaura) at a base pressure of 8.2 × 10^–4^ Pa and a chamber pressure of 0.43 Pa, which were adjusted using an argon (Ar) flow. The substrate was then annealed in a N_2_ atmosphere at 300 °C for 1 h to improve film quality and prevent changes in the film characteristics over time during sensing measurements in the presence of heat. The Au nanosheet was patterned using electron beam lithography. A negative-type electron beam (EB) resist (OEBR-CAN, Tokyo Ohka Kogyo Co.) was spin-coated at 3000 rpm for 60 s on the substrate. The substrate was exposed in a lithography system (F7000S-VD02, Advantest Co.), and the EB resist was developed in an alkali solution (NMD-W, Tokyo Ohka Kogyo Co.) for 60 s. The Au nanosheet was then etched using an Ar dry-etching system (CE-300I, ULVAC Inc.) with the EB resist as a mask. The EB resist was then removed using a piranha solution, and the etched substrate was rinsed with water for 5 min. The electrode patterns were fabricated through the lift-off method using the same EB lithography system. Hexamethyldisilazane (used as a primer; Tokyo Ohka Kogyo Co.) and a positive-type EB resist (ZEP520A-7, Zeon Co.) were spin-coated on the substrate, which was then exposed and developed in a developing solution (ZED-N50, Zeon Co.). Au electrodes (100 nm thickness) with a Cr adhesion layer of 5 nm were deposited via EB evaporation. The substrate was cleaned using *N*,*N*-dimethylethanamide and 2-propanol through ultrasonication for 10 min (5 min per solution).

### Characterization

The morphology of the Au nanosheet was characterized via field-emission scanning electron microscopy (SEM; S-4200, Hitachi), transmission electron microscopy (TEM; H-9500, Hitachi), and energy-dispersive X-ray spectroscopy (EDX; JED-2300T, JEOL). The nanosheet crystallinity and orientation were characterized via nanobeam diffraction (NBD; JEM-ARM200F, JEOL) and electron backscatter diffraction (EBSD; JSM-7001FA, JEOL). X-ray photoelectron spectroscopy (XPS; JPS-9010MC, JEOL) was performed to identify the adsorption states of H_2_S on the Au nanosheet surface.

### Gas-Sensing Setup

The gas-sensing measurement setup is shown in Figure S4a. Dry air was used as the base gas to measure the gas-sensing properties. The concentration of the target gas (H_2_S) and relative humidity were controlled by mixing gases whose flow rates were controlled using flowmeters. All gas-sensing measurements were performed in a closed box, as shown in Figure S4b. We applied a drain voltage of 10 mV using a source meter (2636A, Keithley) and measured the nanosheet resistance. The device temperature was controlled by using an external heater in conjunction with a thermocouple. The sensor response was represented by target-gas-induced changes in resistance normalized by the original resistance Δ*R*/*R*_0_ (%), where *R*_0_ represents the resistance of the sensor established by air (base gas) flow and Δ*R* represents the resistance change following H_2_S (target gas) exposure.

### Breath Measurement

The detailed procedure is explained in the Breath Analysis section. Tedlar bags with a volume of 1 L were used to collect healthy breath samples. H_2_S of different concentrations was injected into the healthy breath samples using a syringe to generate simulated breaths. The healthy and simulated breaths were delivered to the Au nanosheet sensors by using a pump and a flowmeter (Figure 4b).

### Clinical Study Design

We performed exhaled-breath measurements on multiple subjects. All experiments were conducted in compliance with the guidelines of the Research Ethics Committee and reviewed and approved by the University of Tokyo Ethics Review Committee on Experimental Research with Human Subjects (Approval Number: KE22-16). Male subjects with no medical history of chronic cardiovascular, respiratory, or mental disabilities were recruited for participation in this test. Before the trial, the subjects were properly informed and signed a consent form that included consent for photography during the test.

### Grid-Based Projector-Augmented-Wave (GPAW) Calculation

The projector-augmented-wave method was adopted with a cutoff energy of 500 eV. The Perdew–Burke–Ernzerhof version of the generalized gradient approximation was used as the exchange-correlation functional.^51^ For the Au nanosheet system, we utilized six Au atomic layers with face-centered cubic (fcc) lattices with a (111) surface orientation. The positions of the three upper Au atomic layers and S atoms were relaxed during optimization, whereas those of the three lower Au layers were fixed. Convergence was deemed to be achieved when the total force was less than 0.03 eV/Å. A slab with vacuum layers with a thickness of 20 Å was utilized.

## Results and Discussion

### Characterization of Au Nanosheet

Figure 2a presents an optical image of the Au nanosheet with Cr/Au electrodes with a channel width of 2 μm and a channel length of 250 μm. A long channel length was applied to increase the sensor resistance and suppress the influence of parasitic resistance on the sensor response. The actual channel width was confirmed by the SEM image (Figure 2b), which shows that a Au nanosheet with a uniform width of approximately 2 μm was properly formed. Figure 2c presents the cross-sectional TEM image of the Au nanosheet, indicating that a flat Au nanosheet with a thickness of approximately 16 nm along with a 2 nm-thick TiN adhesion layer formed on the SiO_2_/Si substrate. We conducted EDX elemental mapping to verify the elemental distribution in the Au nanosheet and the TiN (actually a little oxidized TiN_*x*_O_*y*_) adhesion layer, and the findings in Figure 2d suggest that the Au and TiN_*x*_O_*y*_ layers are completely discrete. Thus, interdiffusion did not occur during N_2_ annealing at 300 °C. We also conducted NBD, as shown in Figure 2e, to confirm that N_2_ annealing enhanced the crystallinity of the Au nanosheet. A color-inverted image showing all diffraction points with assigned Miller indices is shown in Figure S5. The lattice constant estimated using the assignment of Miller indices was 4.152 Å, which is reasonable for fcc Au.^52^ The diffraction patterns indicated that the Au nanosheet had good crystallinity with the surface orientation being Au{111}. Its crystallinity and surface orientation were confirmed via EBSD analysis. Figure 2f and g present the inverse pole figure (IPF) map and the pole figure (PF) of the Au nanosheet. The Au nanosheet was mostly (111)-oriented in the direction perpendicular to the nanosheet surface, and it did not have an in-plane orientation. Figure 2h depicts the grain size distribution of the Au nanosheet, which was obtained from the EBSD map. The average grain size was approximately 200 nm, which is more than ten times the film thickness. From these results, we concluded that we successfully fabricated Au nanosheets with high crystallinity and few impurities, and their resistance changes caused by H_2_S adsorption on the Au nanosheet surface (i.e., the sensor response) could be measured easily.

### Gas-Sensing Characteristics

**Figure 3:**
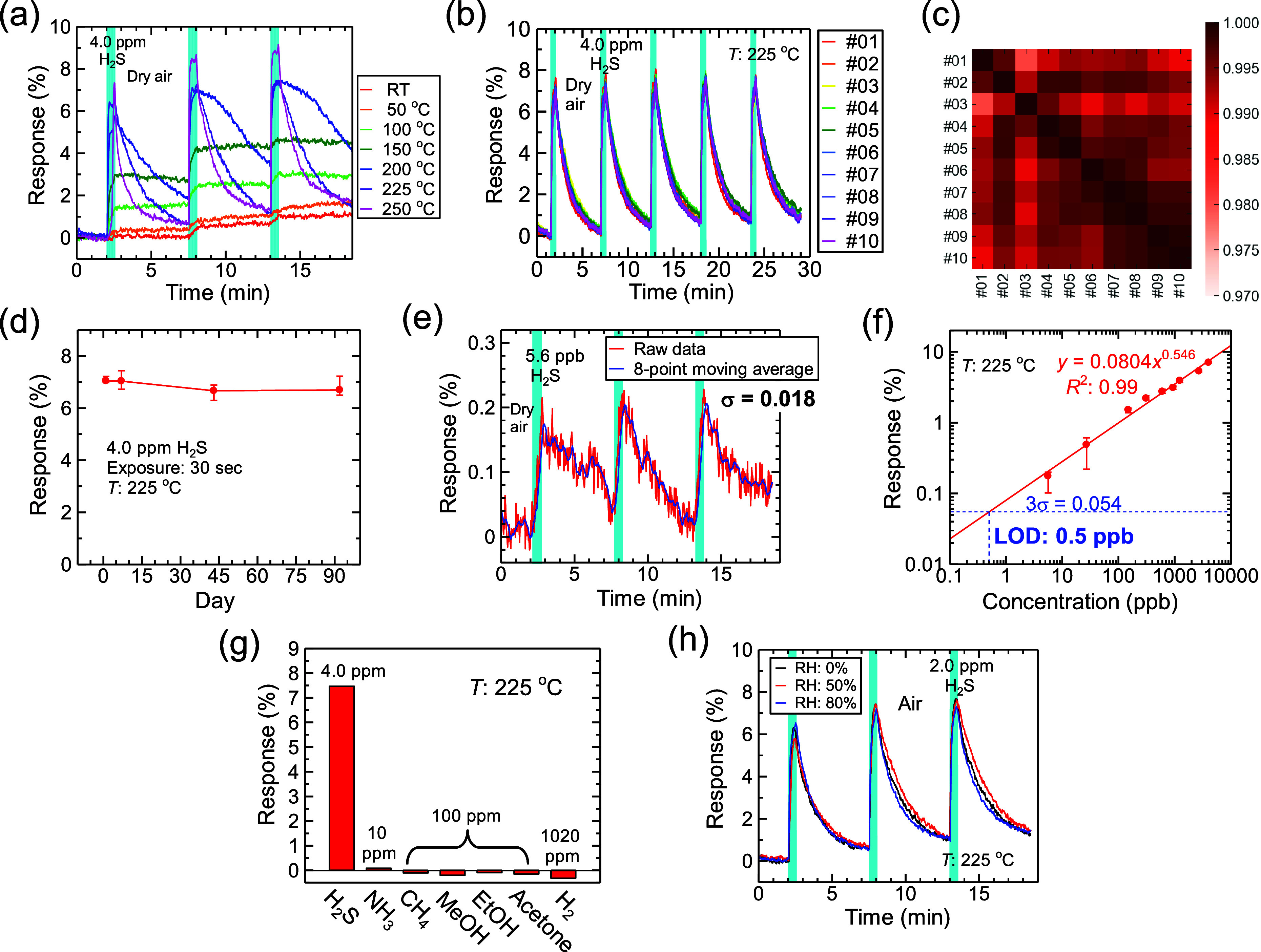
(a) Sensor responses of the Au nanosheet sensor as a function of time at various temperatures. (b) Repeatability characteristics of 10 Au nanosheet sensors exposed to 4.0 ppm of H_2_S gas at 225 °C. (c) Heat map of correlation coefficients between 10 Au nanosheet sensors. (d) Long-term stability of Au nanosheet sensors. (e) Raw sensor response to 5.6 ppb of H_2_S at 225 °C (red line). The blue line is processed by an 8-point moving average. (f) Log–log graph of the relationship between sensor response and H_2_S concentration. (g) Selectivity of the Au nanosheet sensor against interfering gases at 225 °C. (h) Sensor responses to 2 ppm of H_2_S at RH values of 0%, 50%, and 80%.

Figure 3a and Figure S6 show the responses of a Au nanosheet sensor exposed to H_2_S gas at a concentration of 4.0 ppm for 30 s in dry air at different operating temperatures. The sensor exhibited fast response–recovery characteristics above 200 °C and showed little or no recovery at temperatures below 150 °C. H_2_S desorption hardly occurred below 150 °C because of the high binding energy of thiolates to Au (approximately 160 kJ/mol),^53^ while H_2_S desorption and its dissociative adsorption frequently occurred above 200 °C. Here, the sensors were not recovered in the 5 min intervals even at temperatures above 200 °C. We presumed that these incomplete recovery characteristics result from the multiple adsorption sites with different adsorption/desorption time constants. It has been experimentally confirmed that the grain boundaries of the Au NPs are more active sites for CO_2_ reduction than the surfaces.^54^ The DFT calculation has also revealed that the binding energies of CO and COOH intermediates are higher on the Au(110) grain boundary than on the Au(110) surface.^55^ Similarly, it is possible that the binding energy of H_2_S at the grain boundaries of the Au nanosheet is higher, and it takes a much longer time to desorb completely. Figure 3b presents the repeatability characteristics of 10 Au nanosheet sensors operating at 225 °C. The sensors exhibited good repeatability, with response and recovery times of 14 and 240 s, respectively. Here, the response/recovery times were defined by measuring the time required for 90% increase/decrease in resistance relative to the resistance change during H_2_S exposure for 30 s. These times are much shorter than those of other H_2_S sensors using nanoscale Au (Table 1). In addition, the sensor response variability was approximately 0.6%, which is smaller than those of other multiple H_2_S devices, due to the small variability in the original characteristics of the sensors (Table 1 and Figure S7). Figure 3c shows the correlation coefficients between the sensor responses of the 10 Au nanosheets. Here, the correlations of all time-series responses between the two sensors were calculated. The mean correlation coefficient was 0.995, indicating that the behavior of the 10 sensors was almost perfectly proportional. This linear relationship and the small variability in the sensor responses (Figure 3b) verified the uniformity of the Au nanosheet sensors. The long-term stability of the Au nanosheets was studied by measuring the sensor responses to 4.0 ppm of H_2_S, as shown in Figure 3d. Although the sensors were not continuously operated and were kept at room temperature (RT) in the air or a vacuum desiccator (mostly in the air) when not in use, their responses hardly decreased in 92 days. The retention rate was 95%, indicating excellent stability. From the viewpoint of sensor performance, a sufficient sensor response to low concentrations is required for detecting H_2_S in exhaled breath. Thus, we investigated the responses of the Au nanosheet sensor to ppb-level concentrations of H_2_S (5.6 and 27 ppb), as shown in Figure 3e and Figure S8a. Here, the parts per billion-level concentrations of H_2_S were accurately measured using a gas chromatography (GC) system (ODSA-P3-A, Nissha FIS), and the results are shown in Figure S8b. The standard deviation of the noise level σ was 0.018% (red line in Figure 3e), and the averaged sensor response to 5.6-ppb H_2_S was 0.174% in the three cyclic measurements. Since the averaged response value was sufficiently higher than 3σ, the sensor successfully detected concentrations as low as 5.6 ppb H_2_S, which is under the odor threshold of humans (8–13 ppb).^56^ Therefore, the LOD of the proposed and fabricated Au nanosheet sensor is greater than that of a typical human nose. Figure 3f shows the sensor responses as a function of the H_2_S concentration. This log–log graph of concentration from 5.6 ppb to 4.0 ppm shows a linear relationship, with a coefficient of determination (*R*^2^) of 0.99. Therefore, H_2_S concentrations could be estimated accurately by using the sensor responses. From this linear relationship and the standard deviation σ obtained in Figure 3e, an LOD of 0.5 ppb was obtained by using the 3σ method. Figure 3g shows the sensor responses to H_2_S and several interfering gases, namely, ammonia (NH_3_), methane (CH_4_), methanol (MeOH), ethanol (EtOH), acetone, and H_2_, all of which are in exhaled breath.^11,12,57−61^ The response to H_2_S at 4.0 ppm was considerably higher than those to the other gases at concentrations of 10, 100, and 1020 ppm. The raw experimental response data as a function of time are shown in Figure S9. Figure 3h shows the responses to 2.0 ppm of H_2_S at relative humidity (RH) values of 0%, 50%, and 80%. The results affirmed the robustness of the sensors to humidity. Sensor robustness is crucial for breath testing because the RH in exhaled breath can be as high as 95%. The sensing performance of various H_2_S sensors using Au is summarized in Table 1. The fabricated Au nanosheet sensors outperformed the other sensors in terms of response–recovery time, LOD, stability, and variability.

**Table 1:** Summary of Reported H_2_S Sensors Using Au^a^

Structure	OT	*t*_res_/*t*_rec_	Conc.	LOD	Selectivity	Stability	Num.	Resistance variability	Response variability
Au nanowire^38^ (Au NW)	RT	15/30 min	25 ppb	2 ppb	Low against NH_3_	Low (1000% in 5 months)	6	Large (10^10^–10^11^ Ω)	Large (17%/38% at 0.5 ppm)^b^
Au film^35^	RT	10 min/-	120 ppb	-	-	-		Single device	
Au film^62^	265 °C	-/-	1 ppb	1 ppb	-	-	50	Low	Low (5%)
CNT with Au NP^63^	RT	6/10 min	20 ppb	3 ppb	-	High (25% in 6 months)		Assembling bundles (difficult to integrate)	
CNT array with Au NP^27^	RT	5/5 min	20 ppb	3 ppb	Low against humidity	Low	64	Large (1–3 kΩ)	Large (10%/21% at 160 ppb)^b^
Polyaniline NW with Au NP^25^	RT	2/5 min	0.1 ppb	-	High against NH_3_	-		Assembling bundles (difficult to integrate)	
WO_3_ NW with Au NP^64^	350 °C	4/20 min	5 ppm	0.17 ppb	High against CO_2_, NO_2_, acetone	-		Assembling bundles (difficult to integrate)	
ZnO NW with Au NP^65^	RT	-/-	10 ppb	0.5 ppb	-	High in 3 weeks		Assembling bundles (difficult to integrate)	
This work (Au nanosheet)	225 °C	14/240 s	5.6 ppb	0.5 ppb	High against NH_3_, H_2_, humidity, etc.	High (5% in 3 months)	10	Low (2%)	Low (0.6%/7.1% at 4.0 ppm)^b^

a O. T., operating temperature; *t*_res_/*t*_rec_, response time/recovery time; Conc., concentration; LOD, limit of detection; Num., number of sensors.

b The left value indicates the difference between the maximum and minimum responses, and the right value indicates the average response.

### Breath Analysis

**Figure 4:**
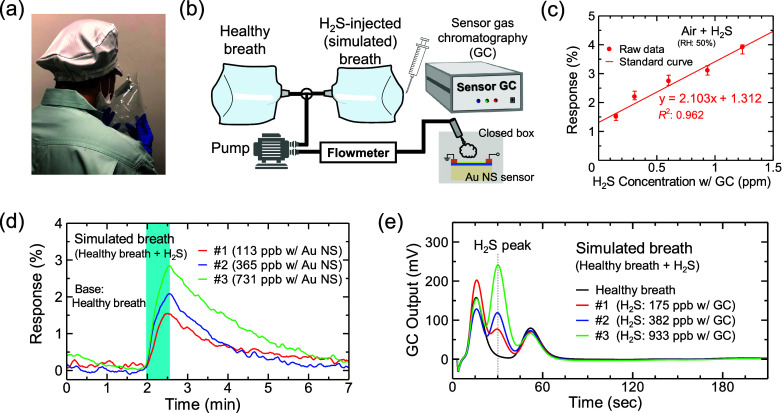
(a) Photo of collection of exhaled breath. (b) Schematic of the simulated breath measurement system. We injected H_2_S of certain concentrations into the healthy exhaled-breath samples to generate simulated breaths. (c) Standard curve of the relationship between sensor response and H_2_S concentration. The H_2_S concentrations were obtained from the GC system. (d) Sensor responses to H_2_S of three concentrations in simulated breaths and concentrations estimated using a standard curve. (e) GC outputs for simulated breaths in (d). The obtained H_2_S concentrations are also shown.

Next, we checked whether H_2_S in exhaled breath could be detected using the Au nanosheet sensors according to the protocol shown in ref (15) because findings showed that the Au nanosheet sensors could detect small amounts of H_2_S without being affected by interfering molecules (Figure 3f and g). The exhaled-breath measurement setup is shown in Figure 4b. We collected healthy exhaled-breath samples in sampling bags, as shown in Figure 4a. The H_2_S concentrations in the exhaled-breath samples were less than the LOD of the GC system, as shown by the black line in Figure 4e. We then injected H_2_S of certain concentrations into healthy breaths to generate simulated breaths. To estimate the H_2_S concentrations in the simulated breaths, we created a standard curve of the relationship between the sensor response and H_2_S concentration. Here, air containing 50% RH was used as a base gas to recreate the humidity condition in the exhaled breath measurement. Figure 4c presents the sensor responses as a function of the H_2_S concentration calibrated using the GC system and the standard curve (the raw response data as a function of time are shown in Figure S10). The results indicated that sub-ppm-level concentrations of H_2_S could be detected. Figure 4d presents the sensor responses to H_2_S in the simulated breaths at three H_2_S concentrations; the peak response values were 1.55%, 2.08%, and 2.85%. The H_2_S concentrations estimated using the standard curve of the Au nanosheet sensors were 113, 365, and 731 ppb. The H_2_S concentrations obtained using the GC system were 175, 382, and 933 ppb (Figure 4e). The other peaks observed in the GC outputs, centered at 17 and 52 s in Figure 4e, corresponded to H_2_ and isoprene, respectively. Both are present in exhaled breath at high concentrations.^12,66^ The H_2_S concentrations estimated using the Au nanosheet sensor were compared with those obtained using the GC system, as summarized in Table 2. The differences seem to result from the incomplete coefficient of determination *R*^2^ (= 0.962) of the standard curve, and this variance may be caused by the unsaturated recovery characteristics (Figure S10) as discussed in the Gas-Sensing Characteristics section. Nevertheless, the observed H_2_S concentrations were comparable to that in halitosis.^9^ Thus, the Au nanosheet sensors can identify H_2_S concentrations in exhaled breath, thus helping to assess the severity of halitosis and diagnose presymptomatic diseases.

**Table 2:** Comparison of Au Nanosheet Sensor (AuNS) and Gas Chromatography (GC) Detection Results from the Same Simulated Breaths

Sample	AuNS sensor (ppb)	GC (ppb)	Δ(GC-AuNS)/GC (%)
#1	113	175	35.4
#2	365	382	4.5
#3	731	933	21.7

### Sensing Mechanism

The gas-sensing mechanism of metal nanosheets is explained by Persson’s model.^67^ Molecular orbitals hybridize with metal orbitals when such molecules are adsorbed on a metal surface; this process forms resonance states characterized by PDOSs. The additional states induced by the adsorbates around the Fermi level of the metal can be the final states of the scattered electrons, resulting in increases in the scattering rate and resistance.

**Figure 5:**
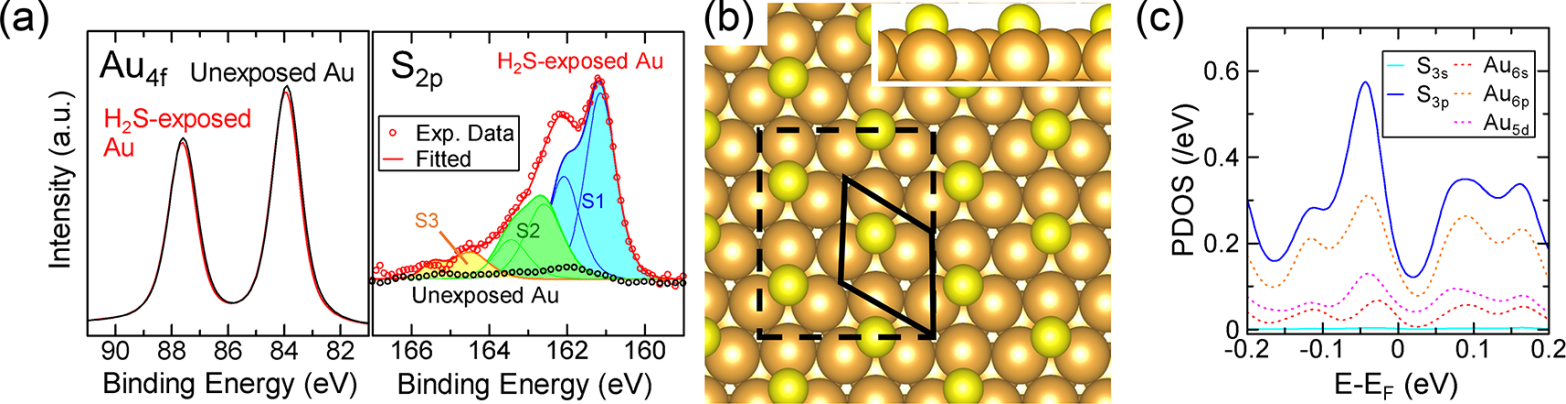
(a) XPS spectra of Au 4f (left) and S 2p (right) from H_2_S-exposed and unexposed Au nanosheets. In the right panel, the best-fitting curve (red line) and the three elemental components (blue, green, and orange) are shown. (b) Optimized structure of the () *R*30° Au(111) surface lattice. The inset shows a side view of the structure. The black solid line represents the real lattice, and the black dashed line represents that used in the DFT calculations. (c) PDOSs of S and Au atoms on Au(111).

We examined the surface states of the Au nanosheets using XPS to verify whether the above-mentioned sensing mechanism could be applied. Figure 5a shows the Au 4f and S 2p spectra of two Au nanosheets; one was exposed to 4.0 ppm of H_2_S for 30 min at 25 °C, and the other was unexposed. The Au 4f_7/2_ spectra indicated binding energy peaks at 84.0 eV for the H_2_S-exposed Au and 83.9 eV for the unexposed Au, which were assigned to Au^0^ (left panel of Figure 5a). Although the peak position of the H_2_S-exposed Au shifted by 0.1 eV and the peak intensity slightly decreased because of the S atoms on the surface, most of the Au existed as Au^0^. The right panel of Figure 5a shows the S 2p spectra; the S 2p_3/2_ peaks at binding energies of 161.1 eV (S1), 162.6 eV (S2), and 164.4 eV (S3) corresponded to three components in the H_2_S-exposed Au, and no S-related peak was detected in the unexposed Au. The S1 component was associated with atomically adsorbed S.^68,69^ The S2 component was ascribed to the S atoms in chemisorbed thiolates, and S3 was ascribed to unbound thiols or disulfide species.^68^ The fitting results suggested that monomeric S atoms were mostly adsorbed on the surface of the Au nanosheet. This adsorption structure was formed by the reaction shown in eq 1, and the S atoms desorbed via the reaction shown in eq 2.^35^
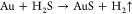
1
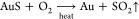
2

Therefore, S atoms chemisorbed on the Au nanosheet surface when the nanosheet was exposed to H_2_S.

Next, we identified the energetic positions of the PDOSs of the S atoms interacting with the Au nanosheet surface. We performed first-principles DFT calculations using the GPAW package.^70^ We applied a () *R*30° Au(111) surface lattice with a surface S coverage θ_S_ of 0.33; the S atoms were adsorbed at 3-fold fcc hollow sites, as shown in Figure 5b. This adsorption structure is typical in S-adsorbed Au(111) systems.^71,72^ In addition, we experimentally observed a {111} surface orientation of the Au nanosheet, as shown in Figure 2e and f. Thus, elucidating the PDOSs of the S atoms using this structure was reasonable. Figure 5c shows the calculated PDOSs of the S atoms. Despite the low PDOSs of the 3s orbitals of the S atoms, relatively large PDOSs of the 3p orbitals of the S atoms existed across the Fermi level. The PDOSs of the Au atoms are also shown in Figure 5c, which indicates that 6s, 6p, and 5d orbitals of the Au nanosheet surface atoms contributed to the covalent bonds of the Au and S atoms. Thus, the PDOSs of the S atoms near the Fermi level increased the resistance of the Au nanosheet. These experimental and computational results provide the first qualitative explanation of the ability of Au nanosheets to detect H_2_S. In the future, we will attempt to quantify the relationship between the amount of H_2_S adsorption and the sensor response.

## Conclusion

We demonstrated Au nanosheet H_2_S sensors with high selectivity, parts per billion-level detection capability, high uniformity, and fast response–recovery characteristics. We optimized the fabrication process of the Au nanosheets to improve sensor response and stability during high-temperature operation. We introduced TiN as an adhesion layer to prevent Au nanosheets from agglomerating on the oxide substrate (SiO_2_). The Au nanosheets were annealed at 300 °C for 1 h to improve their crystallinity; this annealing process reduced the residual resistance components of grain boundary scattering, improved the sensor response, and ensured the thermal stability of the Au nanosheets. The sensors detected H_2_S at concentrations as low as 5.6 ppb, and the estimated LOD was 0.5 ppb. Hence, Au nanosheet sensors are more sensitive than human noses. The sensors were also used to detect H_2_S in the exhaled breaths of simulated patients at concentrations as low as 175 ppb, and they showed sufficient responses, reproducibility, and selectivity against interfering molecules (e.g., H_2_, NH_3_, and alcohols) and humidity. Furthermore, 10 Au nanosheet sensors had very low sensor-to-sensor variability, and the sensor performance was superior to that of other H_2_S sensors by using Au. Finally, we identified the adsorption state of H_2_S on the Au nanosheet surface and calculated the PDOSs of the adsorbed molecules. The results confirmed that monomeric S atoms were mostly adsorbed on the Au nanosheet surface, and the PDOSs of the S atoms were attributed to an increase in the resistance of the Au nanosheet. This Au nanosheet H_2_S sensor, which has uniform sensor characteristics, can be used for facile health checkups using exhaled breath upon its integration into mobile electronic terminals such as smartphones and smartwatches.

## Supporting Information Available

Comparison of adhesion layers, images of Au nanosheet surfaces at different annealing temperatures, the device fabrication process, gas-sensing setup, color-inversed NBD image, temperature dependence of the response, variability of resistances in 10 Au nanosheet sensors, ppb-level detection, raw response data to interfering gases, and raw response data for the standard curve (PDF)
