# Supplementary material for: Detection of PPB-Level H2S Concentrations in Exhaled Breath Using Au Nanosheet Sensors with Small Variability, High Selectivity, and Long-Term Stability
Source: ACS Sens. 2024 Feb 9;9(2):708–16. doi: 10.1021/acssensors.3c01944 (PMC10898455; doi:10.1021/acssensors.3c01944)
Supplement: Supplementary file 1 — se3c01944_si_001.pdf [file se3c01944_si_001.pdf]

Supporting Information: Detection of PPB-Level H_2S Concentrations in Exhaled Breath Using Au Nanosheet Sensors with Small Variability, High Selectivity, and Long-Term Stability

Taro Kato,* Takahisa Tanaka, and Ken Uchida*

Department of Materials Engineering, The University of Tokyo, Tokyo

E-mail: kato@ssn.t.u-tokyo.ac.jp; uchidak@material.t.u-tokyo.ac.jp

Contents

Figure S1. Comparison of influence of adhesion layers on resistivity of Au nanosheet.

Figure S2. Images of Au nanosheet surfaces at different N_2 annealing temperatures.

Figure S3. Schematic of fabrication process of Au nanosheet.

Figure S4. Gas-sensing setup.

Figure S5. NBD image of Figure 2e with color inversion.

Figure S6. Temperature dependence of sensor response of Au nanosheet toward 4.0 ppm H_2S.

Figure S7. Variability of resistances in 10 Au nanosheet sensors.

Figure S8. Sensor response to ppb-level concentrations of H_2S.

Figure S9. Raw experimental response data to interfering gases.

Figure S10. Sensor responses of Au nanosheet sensor to the 5 different concentrations of H_2S for creating the standard curve.

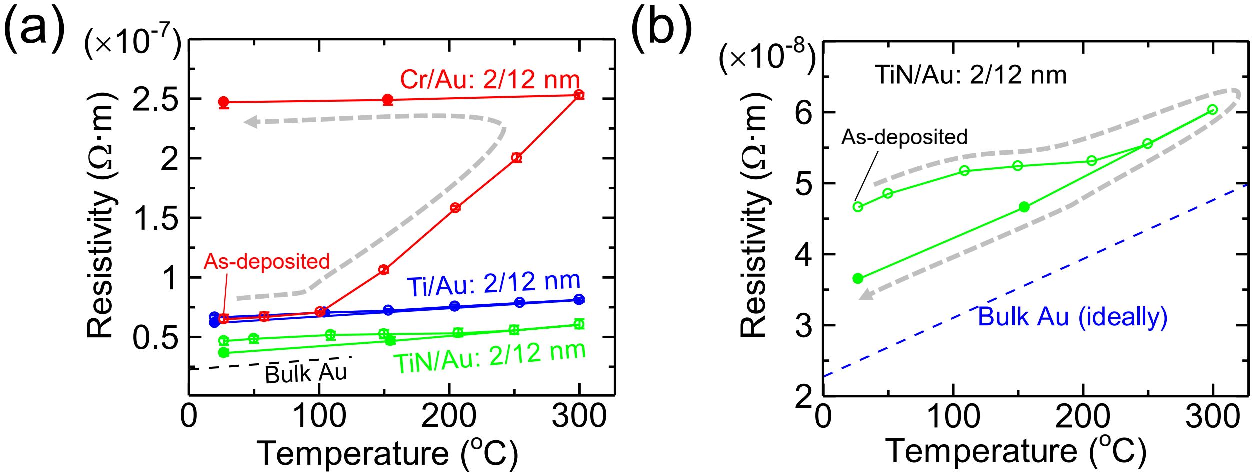

Figure S1: (a) Comparison of temperature dependence of resistivity in 12-nm-thick Au nanosheet with different 2-nm-thick adhesion layers: Cr, Ti, and TiN. (b) Partial enlargement of resistivity data in (a) when TiN adhesion layer is used.

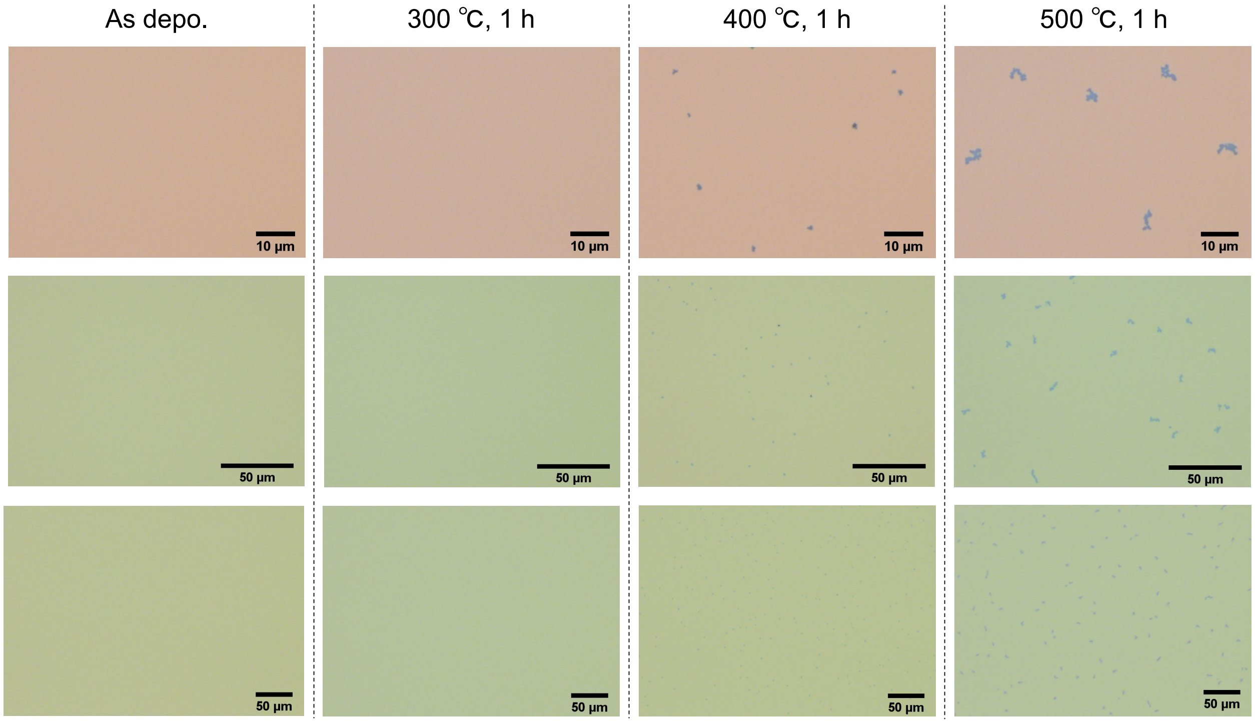

Figure S2: Optical microscope images of Au nanosheet surfaces at different N_2 annealing temperatures. At temperatures above 400 °C, voids formed on Au nanosheets.

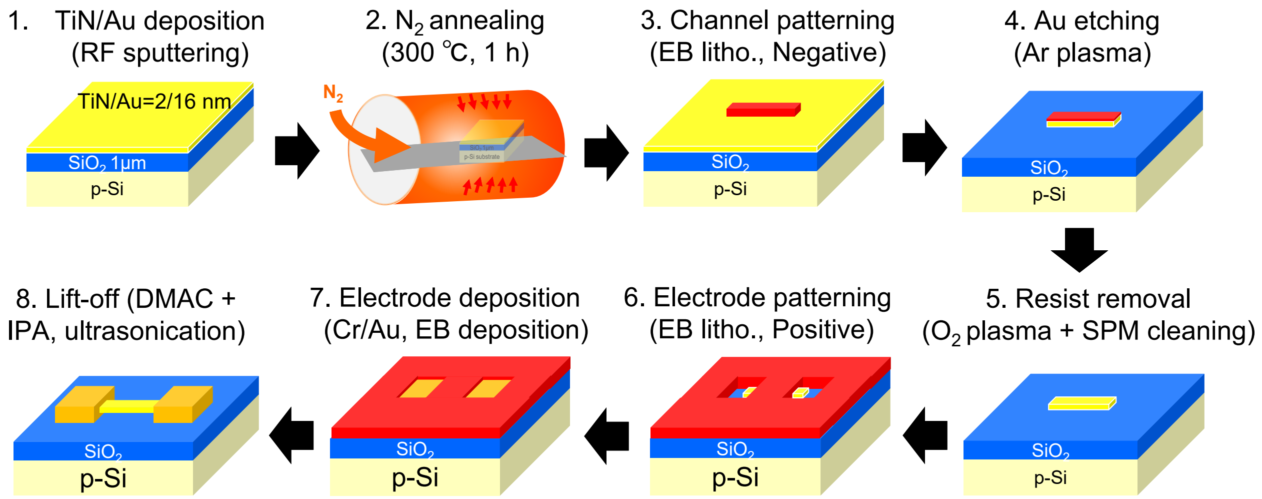

Figure S3: Schematic of fabrication process of Au nanosheet.

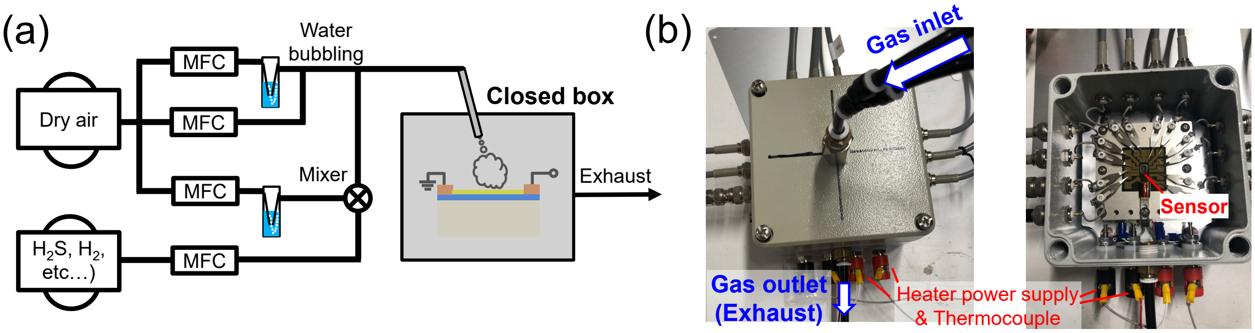

Figure S4: (a) Schematic of gas-sensing measurement setup. (b) Photographs of outside (left) and inside (right) of measurement system.

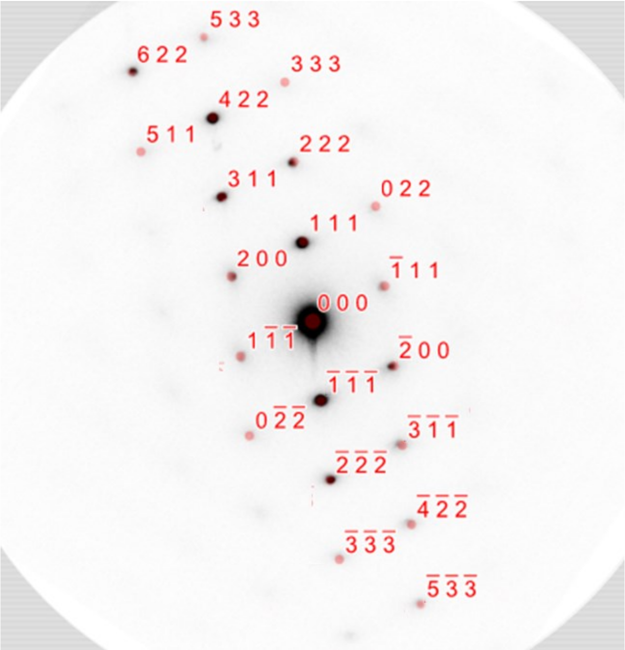

Figure S5: NBD image of Figure 2e with color inversion. Miller indice are assigned to all the diffraction points. Assignment was conducted using SingleCrystal from CrystalMaker®.

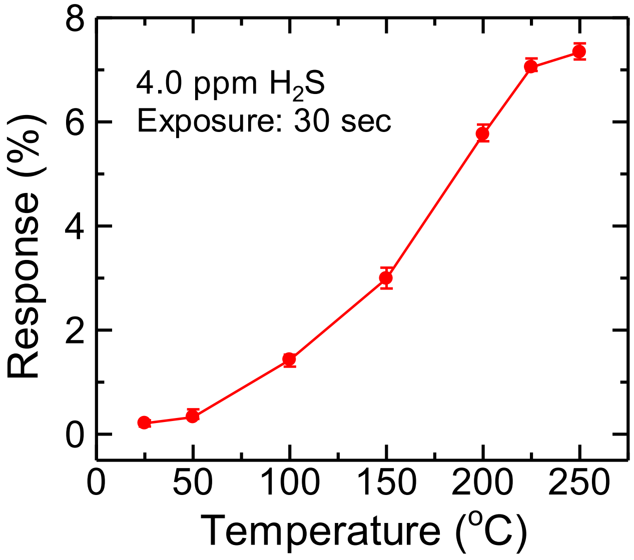

Figure S6: Temperature dependence of response of Au nanosheet sensor to 4.0 ppm H_2S.

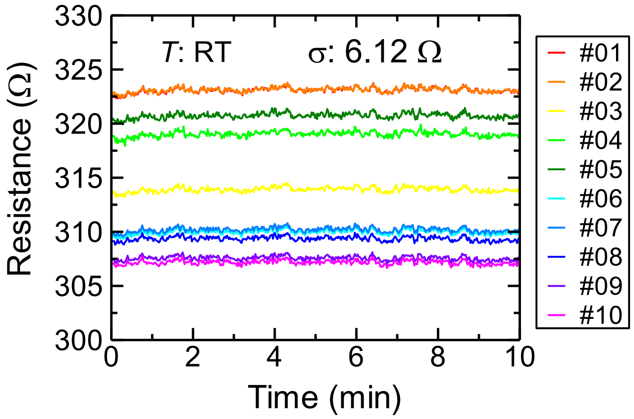

Figure S7: Time characteristics of resistances in 10 Au nanosheet sensors at room temperature. Standard deviation of resistance is 6.12 Ω, which is about 2% of resistance in each sensor.

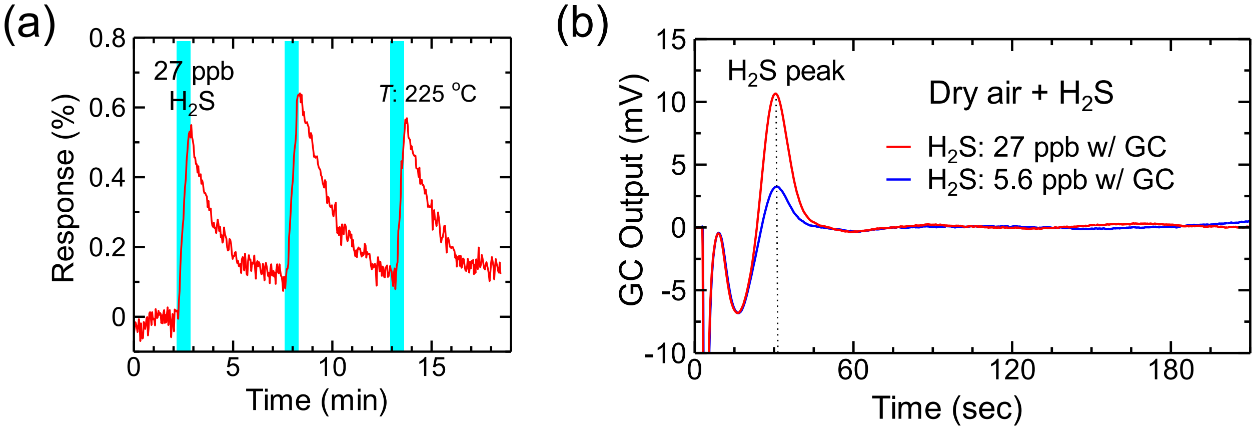

Figure S8: (a) Sensor response to H_2S at a concentration of 27 ppb at 225 °C. (b) Outputs from gas chromatography (GC) to the same H2S gases in (a) and Figure 3e.

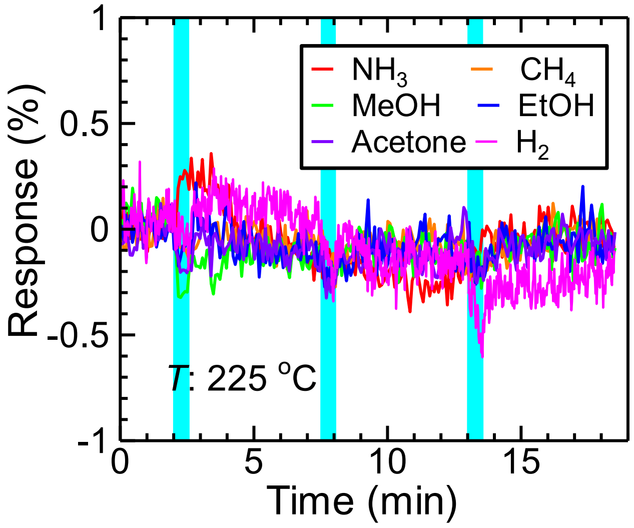

Figure S9: Sensor responses of Au nanosheet sensor to different disturbing gases at 225 °C: 10 ppm NH3 (red), 100 ppm CH4 (orange), 100 ppm methanol (green), 100 ppm ethanol (blue), 100 ppm acetone (purple), and 1020 ppm H2 (pink).

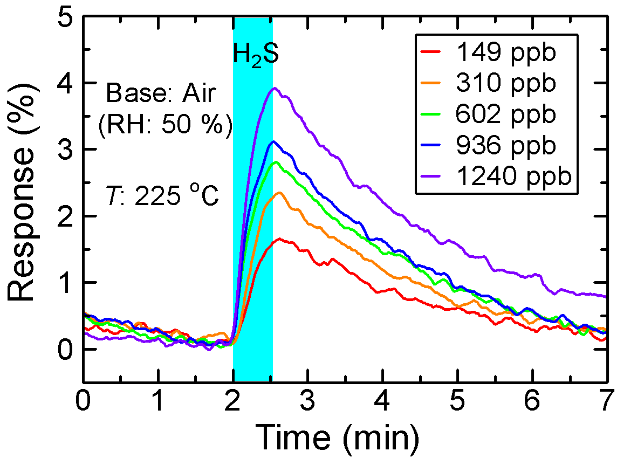

Figure S10: Sensor responses of Au nanosheet sensor to 5 different concentrations of H_2S for creating standard curve. Base gas is air containing relative humidity (RH) of 50%. H_2S gases contain the same humidity.
